# Molecular pathways of pannexin1-mediated neurotoxicity

**DOI:** 10.3389/fphys.2014.00023

**Published:** 2014-02-11

**Authors:** Valery I. Shestopalov, Vladlen Z. Slepak

**Affiliations:** ^1^Department of Ophthalmology, Bascom Palmer Eye Institute, University of Miami Miller School of MedicineMiami, FL, USA; ^2^Department of Cell Biology and Anatomy, University of Miami Miller School of MedicineMiami, FL, USA; ^3^Vavilov Institute of General Genetics, Moscow, Russian Federation, University of Miami Miller School of MedicineMiami, FL, USA; ^4^Department of Molecular Pharmacology, University of Miami Miller School of MedicineMiami, FL, USA; ^5^Neuroscience Program, University of Miami Miller School of MedicineMiami, FL, USA

**Keywords:** hemichannel, neurotoxicity, danger signals, pannexin, neuronal death, inflammasome, calcium, signaling

## Abstract

Pannexin1 (Panx1) forms non-selective membrane channels, structurally similar to gap junction hemichannels, and are permeable to ions, nucleotides, and other small molecules below 900 Da. Panx1 activity has been implicated in paracrine signaling and inflammasome regulation. Recent studies in different animal models showed that overactivation of Panx1 correlates with a selective demise of several types of neurons, including retinal ganglion cells, brain pyramidal, and enteric neurons. The list of Panx1 activators includes extracellular ATP, glutamate, high K^+^, Zn^2+^, fibroblast growth factors (FGFs),pro-inflammatory cytokines, and elevation of intracellular Ca^2+^. Most of these molecules are released following mechanical, ischemic, or inflammatory injury of the CNS, and rapidly activate the Panx1 channel. Prolonged opening of Panx1 channel induced by these “danger signals” triggers a cascade of neurotoxic events capable of killing cells. The most vulnerable cell type are neurons that express high levels of Panx1. Experimental evidence suggests that Panx1 channels mediate at least two distinct neurotoxic processes: increased permeability of the plasma membrane and activation of the inflammasome in neurons and glia. Importantly, both pharmacological and genetic inactivation of Panx1 suppresses both these processes, providing a marked protection in several disease and injury models. These findings indicate that external danger signals generated after diverse types of injuries converge to activate Panx1. In this review we discuss molecular mechanisms associated with Panx1 toxicity and the crosstalk between different pathways.

Connexin and pannexin families of channel proteins connect cells to the environment by forming unpaired half-channels (hemichannels) in the plasma membrane (Bennett and Goodenough, 1978; Jones et al., 1997; Johnson and Owens, 1999). In contrast to gap junctions that mediate direct electrical and metabolic coupling between cells (Goodenough et al., 1980; Draguhn et al., 1998; Bennett and Zukin, 2004), hemichannels support cell-environment communication and paracrine signaling (Jones et al., 1997; Kumar et al., 2004). For example, Pannexin1 (Panx1), which forms predominantly hemichannels, was shown to be involved in paracrine signaling when activated by ATP, UTP, and adenosine. In neurons, Panx1 hemichannels are implicated in electrical communication (Iglesias and Spray, 2012), metabolic autocrine regulation (Kawamura et al., 2010), short-term memory formation (Prochnow et al., 2012), regulation of cell volume during high activity (Wurm et al., 2008, 2010; Li et al., 2012), proliferation and migration of neural stem cells (Wicki-Stordeur et al., 2012), apoptotic “find me” signaling (Chekeni et al., 2010; Sandilos et al., 2012) and possibly other homeostatic functions.

It is known that, under conditions of stress and injury, neural cells rapidly decrease intercellular communication via gap junctions and, instead, switch to hemichannels formed by either connexins or pannexins that communicate with the environment (Melov et al., 2007; Kim et al., 2008; Orellana et al., 2009). These changes are likely modulated by growth factors, such as FGFs (Reuss et al., 2000; Orellana et al., 2009; Garre et al., 2010). Active involvement of hemichannels, particularly Panx1, has been documented in various CNS and PNS pathologies, including hippocampal neuron ischemia (Thompson et al., 2006), retinal ischemia-reperfusion, in human spinal cord injury and thromboembolytic stroke, white matter ischemic injuries, spreading depression, inflammatory enteric colitis, and pain formation (de Rivero Vaccari et al., 2008; Zhang et al., 2008; Abulafia et al., 2009; Domercq et al., 2010; Orellana et al., 2010; Bargiotas et al., 2011; Dvoriantchikova et al., 2012b; Karatas et al., 2013). Experimental data from both pharmacological blockade of the Panx1 channel and genetic ablation of its gene supports a model where Panx1 activation is pivotal for facilitating selective neuronal demise in these pathologies.

Interestingly, Panx1 expression level in retinal ganglion and other neurons is at least 10 times lower than Cx36. However, as indicated above genetic ablation or pharmacologic blockade of Panx1, but not the Cx36 is profoundly neuroprotective in the injured retina. In contrast, the deficiency in functional Cx36 has been liked to secondary degeneration following retinal injury (Striedinger et al., 2005). Taken together, the relatively low expression levels and profound effect of the knockout, indicate that Panx1 activation is particularly neurotoxic (Bargiotas et al., 2011; Dvoriantchikova et al., 2012b).

## Toxicity-associated properties of pannexin-1 channels

**Figure 1:**
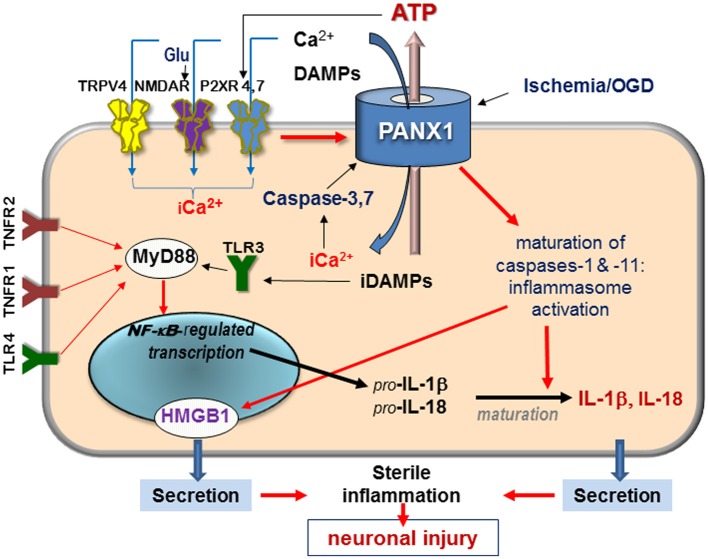
**Schematic diagram of signaling mediated by surface receptors and Panx1—in the injured retina in response to ischemia and extracellular danger signals**. Abbreviations: Glu, glutamate; DAMPs, danger-associated molecular patterns; iCa^2+^, intercellular free calcium; TRPV, transient receptor potential vanilloid, NMDAR, N-methyl-D-aspartate receptor; P2XR, purinergic 2 receptor; TLR, Toll-like receptor, TNFR, tumor necrosis factor receptor; HMGB1, high-mobility group protein B1; red arrows denote activation pathways.

Experimental evidence indicates that unique physiological properties of the Panx1 channel, including permeability to Ca^2+^, ATP, and other small molecules contribute to the pathophysiology of neuronal injury (Bao et al., 2004; Barbe et al., 2006; Locovei et al., 2006a,b; Pelegrin and Surprenant, 2006; Thompson et al., 2006). Unlike connexins, which are closed at physiological concentrations of extracellular Ca^2+^ (Barbe et al., 2006), pannexins remain open and can pass extracellular Ca^2+^ across the plasma membrane (Vanden Abeele et al., 2006). Furthermore, Panx1 hemichannels open in response to elevated intracellular Ca^2+^ (Locovei et al., 2006b), contributing to a rapid post-injury entry of extracellular Ca^2+^ into the cell (“calcium overload”) following various injuries. Indeed, several studies showed that Panx1 activation facilitates Ca^2+^ passage across the plasma membrane (Vanden Abeele et al., 2006; Thompson et al., 2008; Dvoriantchikova et al., 2012b; Weilinger et al., 2013) (Figure 1).

Another immediate consequence of a prolonged activation of Panx1 channel is the efflux of ATP into the extracellular space (Bao et al., 2004; Reigada et al., 2008). Extracellular ATP activates purinergic P2 receptors at the cellular surface, induces IP3-mediated Ca^2+^ release from the endoplasmic reticulum storage and downstream signaling cascades associated with inflammation, and results in neurotoxicity. Compared with connexin, pannexin hemichannels have superior permeability to ATP, thus representing a major release pathway from stressed or injured neurons and, possibly, glia (Iglesias et al., 2009; Iglesias and Spray, 2012; Prochnow et al., 2012; Xia et al., 2012; Dahl et al., 2013). That said, glial cells may also release ATP via the Cx43 hemichannels, as shown by several investigators (Saez et al., 2010; Orellana et al., 2011). This mechanism, however, was questioned by others (Iglesias et al., 2009; Suadicani et al., 2012) because ATP release was disrupted in Panx1-null astrocytes but remained unaffected in Cx43-null astrocytes. Therefore, it is reasonable to suggest that Panx1, which is particularly abundant in several types of CNS neurons (Bruzzone et al., 2003; Dvoriantchikova et al., 2006; Zoidl et al., 2007), is directly responsible for the raise in extracellular ATP and intracellular Ca^2+^, the two major events leading to CNS injury.

In contrast to connexins, Panx1 can be activated by a broad spectrum of extra- and intracellular stimuli. Many of these danger signals are released after injuries and in pathologies and include (in addition to ATP) extracellular K^+^ and Zn^2+^, glutamate, and pro-inflammatory cytokines. Within apoptotic cells, Panx1 is irreversibly activated by proteolysis with caspases 3 and 7 (Bao et al., 2004; Barbe et al., 2006; Brough et al., 2009; Bunse et al., 2009; Orellana et al., 2009; Chekeni et al., 2010). Panx1 is also mechanosensitive and opens by the stretch of the plasma membrane that occurs during changes in osmolarity or mechanical injuries. The result of a prolonged Panx1 activation is massive influx of ions and small molecules into the cell and the efflux of ATP and UTP, as schematically shown in Figure 1.

## Functional interactions with receptors and channels

Key interaction partners for Panx1 include surface receptors and channels, which can be grouped by the following molecular consequences: (i) interactions modulating intracellular Ca^2+^, which include NMDA, P2X, and TRPV4 receptors; (ii) interactions with Ca^2+^-mobilizing G protein-coupled receptors, such as P2Y and PAR1 receptors that mobilize intracellular Ca^2+^ and activate PKC; (iii) interactions leading to ATP/ADP/adenosine signaling that also include purinergic P2X receptors (P2XRs) (Figure 1). Available data suggest that direct binding partners of Panx1 are P2XRs and actin filaments. Functional modulators include NMDA, A1/A2 adenosine receptors, Kvβ 3 potassium channels and fibroblast growth factor-1 (FGF-1) (Bunse et al., 2009; Garre et al., 2010).

Currently, the most established mechanism of cell injury in post-ischemic neurons is Ca^2+^ entry via ligand-gated channels, such as NMDA receptor (NMDAR) and P2XRs (Lazarewicz et al., 1990; Lobner and Lipton, 1993; Sucher et al., 1997; Franke et al., 2006; Hardingham, 2009; Matute and Cavaliere, 2011). However, recent studies indicated that these Ca^2+^ entry mechanisms are significantly facilitated by Panx1 channel activity (Zhang et al., 2005a; Thompson et al., 2008; Orellana et al., 2011; Gulbransen et al., 2012; Weilinger et al., 2013). Thus, according to Thompson and co-authors, Panx1 opening is synergistic with activation of NMDAR and can be facilitated by NMDA or glutamate (Thompson et al., 2008; Weilinger et al., 2013). This group also recently showed that both NMDAR antagonists, attenuated currents carried by Panx1 and the SFK-Panx1 interfering peptide significantly reduced anoxic depolarization, and Ca^2+^ influx in OGD (Weilinger et al., 2012). According to their model, Panx1 activation occurs downstream of NMDAR and is mediated by Src family kinases. If this model is correct, Ca^2+^ overload after ischemic injury occurs via both NMDAR channel activity and the opening of Panx1. Thus, extracellular glutamate is one of several major pathological factors can facilitate Panx1overactivation and the ensuing neurotoxicity.

Ca^2+^ can also enter cells via the purinergic P2XRs activated by extracellular ATP, and via transient receptor potential vanilloid receptors (e.g., TRPV4) activated by mechanical stress (Mochizuki et al., 2009; Ryskamp et al., 2011). Panx1 can facilitate both these mechanisms to the point that transient extracellular Ca^2+^ changes are converted into the pathogenic overload. Furthermore, ATP can be released via Panx1, further activating P2XRs in paracrine or autocrine fashion (Figure 1). Functional interaction between P2X7 receptor and Panx1 was first reported to occur in macrophages by Pelegrin and Surprenant (2006), who suggested on the basis of co-immunoprecipitation of ectopically expressed Panx1 and P2XR isoforms that these two proteins can interact directly. P2X7-dependent opening of the Panx1 channel in response to ATP has been demonstrated in diverse pathological settings, prompting researchers to name Panx1 “the pore-forming unit of P2X7R-death complex” (Locovei et al., 2007; Iglesias et al., 2008). In the CNS, a similar role was suggested for Panx1 complex with P2X4R (de Rivero Vaccari et al., 2012), which is expressed more abundantly than P2X7R in neurons of the spinal cord and in retinal ganglion cells. Physical interaction between Panx1 and P2XRs 2,3,4, and 7 were shown by co-immunoprecipitation in rat pituitary cells (Li et al., 2011). Studies showed that Panx1-mediated ATP release in certain cell types can also occur in response to activation of metabotropic P2Y1 and P2Y2 receptors (Locovei et al., 2006b; Zhang et al., 2012).

Interactions between TRPV4 channel and Panx1 hemichannel were reported recently in the airway and ocular lens epithelium (Seminario-Vidal et al., 2011; Shahidullah et al., 2012). These results indicate that TRPV4 and associated Rho activity can transduce cell membrane stretch to Panx1, which releases ATP (Iglesias et al., 2008). As was suggested recently (Krizaj et al., 2013), this novel model can provide feasible explanation for severe Ca^2+^ disregulation observed in retinal disorders linked to mechanical stretch of the retina, such as intraocular pressure-induced primary glaucoma. Importantly, TRPV4 activation was shown to predispose RGCs to death via Ca^2+^-dependent pro-apoptotic signaling pathways (Ryskamp et al., 2011). Additional binding partners and modulators of Panx1 activity are A1 and A2 adenosine receptors (Kawamura et al., 2010), Kvβ 3 potassium channels (Bunse et al., 2009), FGF-1 (Garre et al., 2010), actin microfilaments (Bhalla-Gehi et al., 2010; Bao et al., 2012), and thrombin-specific protease-activated receptor 1 (PAR-1) (Godecke et al., 2012). The significance of these recently detected interactions remains to be determined.

Interestingly, in many cell types expressing P2XRs, receptor-induced opening of the Panx1 channel is a relatively late event that is preceded by the rapid opening of the receptor channel and occurs after a prolonged agonist application. Prior to the discovery of pannexins, this delay was explained as a slow development of a large pore via progressive dilation of P2XRs themselves upon continuous/repetitive application of agonist (Innocenti et al., 2004; Gourine et al., 2007). However, later studies showed that Panx1 is required for generation of the characteristic large current (~500 pS) and permeability to molecules up to 900 Da (Pelegrin and Surprenant, 2006, 2009; Locovei et al., 2007; Iglesias et al., 2008). In the complex with Panx1 channel, stimulation of P2XRs leads to significantly higher currents compared to the activation of either P2XRs, NMDAR, or TRPV4 alone. These experiments allowed researchers to separate receptor activation and Panx1 opening events (Iglesias et al., 2008). Physiologically, the delayed Panx1 opening may provide for a tighter control of this large cell-permeating pore, so that only abnormally high level of activation would amplify Ca^2+^ uptake and ensuing neurotoxicity (Locovei et al., 2007). Indeed, several lines of experimental data indicate that ischemia-mediated Panx1 opening also occurs 10–20 min after the onset of ischemic conditions (Thompson et al., 2006; Zhang et al., 2008; Dvoriantchikova et al., 2012b; Weilinger et al., 2012). This delay could be attributed to lagging development of conditions favoring Panx1 opening, such as anoxic depolarization and/or accumulation of agonists (danger factors).

## Neurotoxic signaling mechanisms downstream of Panx1

What are the specific mechanisms of cell death downstream of P2XR(4,7)–Panx1 complex? The most thoroughly studied mechanism involves intercellular ionic dysbalance, mostly Ca^2+^ overload and the subsequent activation of Ca^2+^-dependent proteases including calpain and pro-apoptotic caspases (Dvoriantchikova et al., 2012b; Paramo et al., 2013; Weilinger et al., 2013) (Figure 1). The P2XR(4,7)–Panx1 complex has also been reported to activate several enzymatic pathways known to damage neuronal cells. The first one acts through the activation of ROS-producing enzyme NADPH oxidase (Seil et al., 2008; Barakat et al., 2012; Choi et al., 2012; Dvoriantchikova et al., 2012a). The second mechanism links P2XR(4,7)–Panx1 overactivation with a relatively new neurotoxic pathway, activation of the inflammasome (de Rivero Vaccari et al., 2012). Inflammasome is an intracellular macromolecular complex responsible for proteolytic processing and release of inflammatory interleukins IL-1β and IL-18 (Pelegrin and Surprenant, 2006; Kanneganti et al., 2007). Inflammasome was first characterized in macrophages and more recently in glia and neurons (Silverman et al., 2009). Activation of caspase-1, the principal component of the complex, was shown to be dependent upon Panx1 in both neuronal and glial cell types (Kanneganti et al., 2007; de Rivero Vaccari et al., 2009; Silverman et al., 2009) but is redundant in macrophages (Pelegrin et al., 2008; Qu et al., 2011). The role of inflammasome activation in human pathologies those affecting the CNS have been extensively reviewed elsewhere (Bernier, 2012; Dahl and Keane, 2012; Strowig et al., 2012; Fleshner, 2013), here we will cover this topic very briefly. Production of interleukins IL-1β and IL-18 depends on: (1) transcriptional activation of the interleukin gene and accumulation of the IL precursors; (2) proteolytic activation of caspase-1 within the inflammasome that is required to processes them into mature forms.

The pathway for transcriptional activation of the IL-1β and IL-18 genes requires activity of surface receptors, including tumor necrosis factor (TNFR) and Toll-like (TLR) receptors, and recruitment of the adapter protein MyD88, which is followed by activation of transcription factor NF-κB (Figure 1) (de Rivero Vaccari et al., 2008; Silverman et al., 2009). Parallel maturation of cytokines appears to be a crucial step in the neurotoxic pro-inflammatory program. This step requires Panx1 (Pelegrin and Surprenant, 2007; Dvoriantchikova et al., 2012b), and is independent on TLR activation (Kanneganti et al., 2007). The inflammasome is rapidly activated in the CNS in sterile (non-microbial) conditions in response to extracellular ATP and other DAMPs (Maher, 2009; Lamkanfi, 2011; Ayna et al., 2012; Riteau et al., 2012). It was also recently shown to be involved in the release of the pro-inflammatory nuclear protein high-mobility group B1 (HMGB1, or “alarmin”) (Karatas et al., 2013; Li et al., 2013).

In the model of retinal ischemia-reperfusion, Panx1-mediated neurotoxicity correlated with robust activation of endogenous, neuronal inflammasome and the release of IL-1β in the ganglion cell layer, the site of major damage (Dvoriantchikova et al., 2012b). According to Pelegrin and co-authors, blocking the Panx1 channels with siRNA, mimetic peptide or carbenoxolone in macrophages prevented large pore formation, caspase-1 activation, and processing/release of IL-1β (Pelegrin and Surprenant, 2006; Pelegrin, 2008). The processing and release of IL-1β in the post-ischemic retina are significantly attenuated by Panx1 knockout (Bargiotas et al., 2011; Dvoriantchikova et al., 2012b). Similarly, experimental blocking of the inflammasome in the CNS through pharmacological inactivation or genetic ablation of either P2X7 receptor, Panx1, or any major component of the inflammasome complex, including caspase-1, NALP, or ASC proteins, had exactly same effect and similar degree of neuroprotection (Zhang et al., 2005a; Arai et al., 2006; de Rivero Vaccari et al., 2009; Domercq et al., 2010). Striking similarity in the degree of neuroprotection upon inactivation of either Panx1 or inflammasome was observed in mouse models of enteric colitis (Gulbransen et al., 2012). Intriguingly, recent studies linked inflammasome activation with the onset of pyroptosis, an inflammatory form of programmed cell death (Franchi et al., 2012; Strowig et al., 2012). Another new non-apoptotic mechanisms of cell death that have been recently linked with ischemic retinal and brain injury, is necroptosis (Rosenbaum et al., 2010; Meloni et al., 2011). However, despite the profile of Panx1-mediated neurotoxicity matches that observed in necroptosis, no correlation between Panx1 activity and necroptosis marker RIP3 has been reported so far.

In addition to interleukin secretion, another pathway, the inflammasome-dependent release of nuclear protein alarmin/HMGB1 can facilitate neurotoxicity indirectly via glia-mediated toxicity. This novel pathway has been recently demonstrated in both inflammatory and neuronal cells (Willingham et al., 2009; Lamkanfi et al., 2010). Initially thought to be released from necrotic or injured cells, massive HMGB1 release has been shown in live brain neurons affected by spreading depression (Karatas et al., 2013), but the mechanism of such release remains poorly characterized. The release of alarmin from live cells is triggered by a spectrum of distinct cytokines and/or danger signals produced during CNS stress or injury, and is recently shown to be controlled by Panx1 (Yang et al., 2010; Karatas et al., 2013). Furthermore, Karatas and coauthors showed that alarmin binds to glial RAGE receptors leading to activation of astroglial NF-κB pathway, which is one of the most potent source of glial neurotoxicity in CNS disorders (Bales et al., 1998; Brambilla et al., 2009, 2012; Dvoriantchikova et al., 2009; Barakat et al., 2012).

The Panx1 channel can provide the gateway for DAMPs/PAMPs to enter the cell and activate intracellular TLRs, such as TLR3, which has been implicated in several neurodegenerations, including glaucoma (Nowak and Davies, 2004; Zhang et al., 2005b; Shiose et al., 2011). These intracellular TLRs receptors signal to NF-κB and are expressed by both glial and microglial cells. These experiments demonstrated that ATP-induced Panx1 channel opening provides an entry pathway for bacterial danger signals into the cytosol, where they facilitated the cryopyrin-dependent activation of caspase-1 (Kanneganti et al., 2007). When stimulated by ATP activation of P2X7R, Panx1 was essential for proteolytic cleavage of caspase-1 and the subsequent maturation and release of its substrate, IL-1β [24, 46, 47]. At the same time, the opening of Panx1 hemichannels was shown to be independent of TLR activation [42], indicating that Panx1 acts in parallel, not downstream of TLRs.

## The role of Panx1 in neurotoxic glia-neuron interactions

How does activation of the ubiquitously expressed Panx1 result in selective injury and death of distinct neuronal types? Such selective vulnerability is consistent with high levels of Panx1 expression in certain neuronal sub-populations, for example RGCs in the retina and hippocampal pyramidal neurons (Dvoriantchikova et al., 2006; Krizaj et al., 2013). An interesting model that proposed a role of activated glia in triggering death of such sub-populations was suggested by Orellana and co-authors. Using an *in vitro* cell culture approach, they showed that neuronal demise is triggered by synergistic action of high extracellular glutamate and ATP on neuronal Panx1 channel (Orellana et al., 2011). The mechanism of massive efflux of astroglial glutamate and ATP in their model was initiated through the release of TNFα and IL-1β cytokines from activated microglial cells. Additional studies are required to test whether this mechanism of action occurs *in vivo*.

Our model summarizing mechanisms underlying Panx1-mediated pathophysiology is depicted in Figure 1. As mentioned above, Panx1 is activated by diverse signaling pathways. In pathological conditions, Panx1 activation parallels transcriptional activation of the TLR-MyD88-NF-κB pathway to synergistically facilitate production and secretion of pro-inflammatory interleukins IL-1β and IL-18. This model implies that transcriptional induction of MyD88-NF-κB by the upstream signaling requires synchronization with activation of Panx1 to achieve a successful cytokine release. Thus, activation of NF-kB-regulated gene transcription downstream of Toll-like and TNF receptors occurs in parallel with the activation of P2X receptors and Panx1, thereby coordinating gene transcription with proteolytic processing of interleukins.

## Panx1 as a pharmacological target

Blockers of Panx1 channel such as probenecid, mefloquine, and carbenoxolone (Iglesias et al., 2008; Silverman et al., 2008) have been used for several decades to treat gout, gastric ulcer and infection diseases such as malaria (Doll et al., 1965; Trenholme et al., 1975; Silverman et al., 2008). Unfortunately, these compounds are not highly selective for Panx1 as they also block other channels, including connexins. Side effects of these drugs include pain, severe muscle weakness, mood changes, seizures, psychiatric adverse reactions, and hypertension, which limits their pharmacological potential. As an alternative, many laboratories successfully utilized panx^10^ anti-peptide to block Panx1 function in experimental settings, however, specificity of this reagent has also been disputed (Dahl, 2007). Development of new Panx1 inhibitors with improved pharmacological characteristics and selectivity will undoubtedly benefit investigators and physicians looking to curb Panx1-mediated neurotoxicity in human pathologies.

## Conclusions

The unique properties of Panx1 channels contribute to the profound neurotoxicity associated with this channel activation during retinal and brain ischemia. The combination of high permeability to ATP, ions, and other molecules, and activation by diverse injury-induced DAMPs, makes Panx1-expressing neurons highly sensitive to mechanical and ischemia injuries. Furthermore, Panx1 works synergistically with other neurotoxic pathways, such as the TLR/TNF receptors-NF-κB axis facilitating pro-inflammatory cytokine and HMGB1 in many cell types. The involvement of Panx1 in multiple neurotoxic pathways, as well as its proven “druggability” make it a promising target for therapies of progressive neurodegenerations of the retina or brain.

### Conflict of interest statement

The authors declare that the research was conducted in the absence of any commercial or financial relationships that could be construed as a potential conflict of interest.
